# Household Transmission of Human Adenovirus Type 55 in Case of Fatal Acute Respiratory Disease 

**DOI:** 10.3201/eid2509.181937

**Published:** 2019-09

**Authors:** Shuping Jing, Jing Zhang, Mengchan Cao, Minhong Liu, Yuqian Yan, Shan Zhao, Na Cao, Junxian Ou, Kui Ma, Xiangran Cai, Jianguo Wu, Ya-Fang Mei, Qiwei Zhang

**Affiliations:** Southern Medical University, Guangzhou, China (S. Jing, J. Zhang, Y. Yan, S. Zhao, N. Cao, J. Ou, Q. Zhang);; Guangdong Provincial Key Laboratory of Tropical Disease Research , Guangzhou (S. Jing, J. Zhang, Y. Yan, S. Zhao, N. Cao, J. Ou, Q. Zhang);; Anqing Center for Disease Control and Prevention, Anhui, China (M. Cao, M. Liu); Jinan University, Guangzhou, China (K. Ma, X. Cai, J. Wu, Q. Zhang); Umeå University, Umeå, Sweden (Y.-F. Mei)

**Keywords:** acute respiratory disease, household transmission, fatality, human adenovirus type 55, adenoviruses, viruses, China

## Abstract

We identified a case of fatal acute respiratory disease from household transmission of human adenovirus type 55 (HAdV-55) in Anhui Province, China. Computed tomography showed severe pneumonia. Comparative genomic analysis of HAdV-55 indicated the virus possibly originated in Shanxi Province, China. More attention should be paid to highly contagious HAdV-55.

Human adenoviruses are associated with mild and acute respiratory infections, depending on the virus type and host immunity. Human adenovirus type 55 (HAdV-55) (*1*), formerly known as HAdV-11a (*2*), is a reemergent respiratory pathogen that has caused severe pneumonia outbreaks in military and civilian populations in Europe and Asia (*2**–**7*). However, household transmission of HAdV-55 is rarely reported. We report a case of household transmission of HAdV-55 involving 3 confirmed adult cases with 1 death. Epidemiologic, clinical, and laboratory investigations, along with whole-genome sequencing, elucidate the disease progression and the pathogen origin.

During April 1–May 5, 2012, 7 household members (5 males and 2 females; 3 children and 4 adults) in Anhui Province, China, sequentially experienced influenza-like symptoms, including fever, productive cough, fatigue, pharyngalgia, dyspnea, and other symptoms. The youngest patient was 4 months of age, the oldest, whom we refer to as AQ-1, was a 55-year-old man. The family lived together near a farm in a house with poor sanitary and ventilation conditions. 

The first onset of acute respiratory disease (ARD) occurred on April 1, when the index case, a 4-year-old granddaughter of AQ-1, had a febrile respiratory infection with cough. Three days later, AQ-1’s grandson, 1 year of age, displayed similar symptoms. On April 9 and 11, AQ-1’s daughter, 28 years of age, and another grandson, 4 months of age, both had influenza-like symptoms. On April 14, AQ-1 had a fever, chills, and lumbago. He was admitted to the hospital on April 14 where clinicians diagnosed pneumonia. AQ-1 had close contact with his sick grandsons and granddaughter and had not been out of the house during the month he cared for them. 

While hospitalized, AQ-1 had bilateral pneumonia seen on chest computed tomography (CT), a temperature of 41.0°C, and low total leukocyte (3.63 × 10^9^/L) and platelet (42 × 10^9^/L) counts. AQ-1 sustained high fever and yellow phlegm despite antiinflammatory and antiviral treatment, including levofloxacin, piperacillin sodium, tazobactam sodium, and ribavirin. 

On April 24, AQ-1 had indications of severe pneumonia, including respiratory failure, hypoxemia, double lung rales, and a mass of shadows visible on chest CT. In addition, he had indications of liver damage and multi-organ failure. Transverse chest CT images demonstrated increased areas of patchy shadows and consolidation in both lungs compared to CT images from April 22, indicative of disease progression (Appendix Figure 1).

AQ-1 died on April 27, 3 days after onset of respiratory failure, and 13 days after his illness began. On the same day, his 20-year-old son, AQ-2, and 31-year-old nephew, AQ-3, who had taken care of AQ-1 for 5 days, also exhibited symptoms of influenza-like illness. Both were hospitalized and had normal chest CT scans, but AQ-2’s leukocyte count was 5.4 × 10^9^/L and AQ-3’s was 6.7 × 10^9^/L. After antiinflammatory and antiviral treatment, including vitamin C, sulbactam, amoxicillin, amikacin, cefoperazone, ribavirin, and oseltamivir, they recovered and were discharged on May 5 (Figure). 

**Figure:**
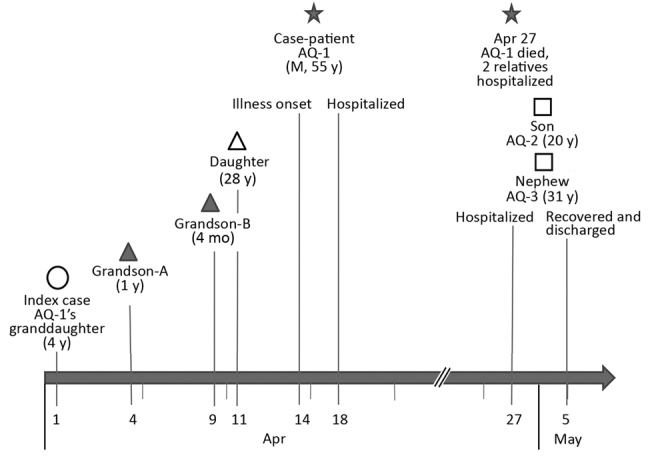
Timeline of patients’ illness onset in a household cluster of acute respiratory disease from human adenovirus 55, Anhui Province, China, 2012. The star indicates AQ-1, the case described in this study. Case relationships to AQ-1 are indicated along with their ages at the date of their illness onset.

We tested endotracheal aspirates from AQ-1 and throat swabs from AQ-2 and AQ-3 for influenza A and B viruses, severe acute respiratory syndrome coronavirus, human metapneumovirus, rhinoviruses, parainfluenza viruses 1–4, and HAdVs by real-time PCR. Only adenovirus was strongly positive for all 3 patients. Testing for antibodies against *Mycoplasma pneumoniae*, *Mycobacterium tuberculosis* *Treponema pallidum*, hepatitis B and C viruses, and HIV, were all negative. After treatment, samples from AQ-2 and AQ-3, were negative for adenovirus by PCR.

We isolated AQ-1’s adenovirus in culture and sequenced the genome (GenBank accession no. KP279748). Sequences for the hexon, penton base, and fiber genes were identical to those previously reported for HAdV-55. Phylogenetic analysis showed that the 3 isolates clustered closely with other strains from China (Appendix Figure 2). The genome of AQ-1’s strain had the highest nucleotide identity (99.951%) with QZ01_2011, an isolate from a military trainee in Shanxi Province, China. The second highest identity (99.948%) was with QS-DLL_2006, which caused a fatal ARD outbreak in a senior high school in Shaanxi Province, China (*1**,**8*) (Appendix Table). We hypothesize the strain infecting AQ-1 and his family originated from Shanxi Province.

In this household transmission of ARD, the index case was a probable case because no specimens were collected to confirm virologic identification. From the timeline of illness onset in this household cluster of ARD cases (Figure), we suspect that the pathogen spread rapidly among the children and further circulated in adults who had close contact with infected children and one another.

HAdV-55 contains a 97.4% genome of HAdV-14 and a hexon from HAdV-11 (*1*). Since 2006, HAdV-14 has caused severe ARD in America, Europe, and Asia (*8*,*9*), with high hospitalization (38%) and case-fatality (5%) rates (*10*). Because the risk for infection among the close contacts may rise, more attention should be paid to these highly contagious pathogens.

Appendix Additional information on household transmission of human adenovirus type 55 in a case of fatal acute respiratory disease.
