# Supplementary material for: Household Transmission of Human Adenovirus Type 55 in Case of Fatal Acute Respiratory Disease
Source: Emerg Infect Dis. 2019 Sep;25(9):1756–8. doi: 10.3201/eid2509.181937 (PMC6711205; doi:10.3201/eid2509.181937)
Supplement: Appendix — Additional information on household transmission of human adenovirus type 55 in a case of fatal acute respiratory disease. [file 18-1937-Techapp-s1.pdf]

Article DOI: https://doi.org/10.3201/eid2509.181937

# Household Transmission of Human Adenovirus Type 55 in Case of Fatal Acute Respiratory Disease

## Appendix

**Appendix Table.** Comparison of mutations across genomes of 3 human adenovirus-55 isolates, China*

| | | | | | Amino acid substitution | |
|---|---|---|---|---|---|---|
| | | | Nucleotide mutation | | QZ01_2011/QS-DLL_2006 | |
| Region | Position | Gene | QZ01_2011 | QS-DLL_2006 | Nonsynonymous | Synonymous |
| E1B | 1721 | 20.8 kDa protein | NA | T→A | –/L→I | –/– |
| E1B | 2170 | 54.7 kDa protein | NA | C→T | –/P→S | –/– |
| IX | 3685 | pIX | T→C | T→C | –/– | A→A/A→A |
| | 3900 | pIX | C→T | NA | S→F/– | –/– |
| E2B | 7863 | DNA polymerase | T→C | T→C | –/– | P→P/P→P |
| L1 | 11623 | 43.9 kDa protein | C→G | C→G | –/– | L→L/L→L |
| | 13609 | poly(A) | ▲A | ▲A | –/– | –/– |
| L2 | 14258 | penton | A→G | A→G | –/– | L→L/L→L |
| | 16581 | pV 40.1 kDa protein | C→G | C→G | –/– | V→V/V→V |
| | 17310–17311 | poly(A) | ▼AA | ▼AA | –/– | –/– |
| | 17328 | poly(A) | ▲A | ▲A | –/– | –/– |
| L3 | 17914 | pVI 26.6 kDa protein | A→C | A→C | H→N/H→N | –/– |
| E2A | 22846 | DNA binding protein | NA | C→A | –/– | –/L→L |
| L4 | 24680 | 91 kDa protein | G→A | NA | S→N/– | –/– |
| | 25509 | 91 kDa protein | NA | G→A | –/– | L→L/L→L |
| E3 | 28827 | 20.3 kDa protein | NA | A→G | –/– | K→K/K→K |
| | 29461 | poly(T) | ▼T | ▼T | –/– | –/– |
| L5 | 31392 | fiber | A→C | NA | T→P/– | –/– |
| E4 | 31874 | ORF6/7 | G→T | NA | –/– | I→I/– |
| NA | 34567 | NCR | G→A | G→A | –/– | –/– |
| NA | 34573 | NCR | ▲G | ▲G | –/– | –/– |

*Genomes of HAdV-55 isolates AQ-1_2012 (Anhui Province), QZ01_2011 (Shanxi Province), and QS-DLL_2006 (Shaanxi Province) analyzed with respect to base substitutions and insertions and deletions, using isolate AQ-1_2012 as reference. Nucleotide and amino acid sequence changes are noted, along with their genome locations and coding regions. NA, not applicable; NCR, non-coding region; ORF, open reading frame; ▼, insertion; ▲, deletion; –, no change.

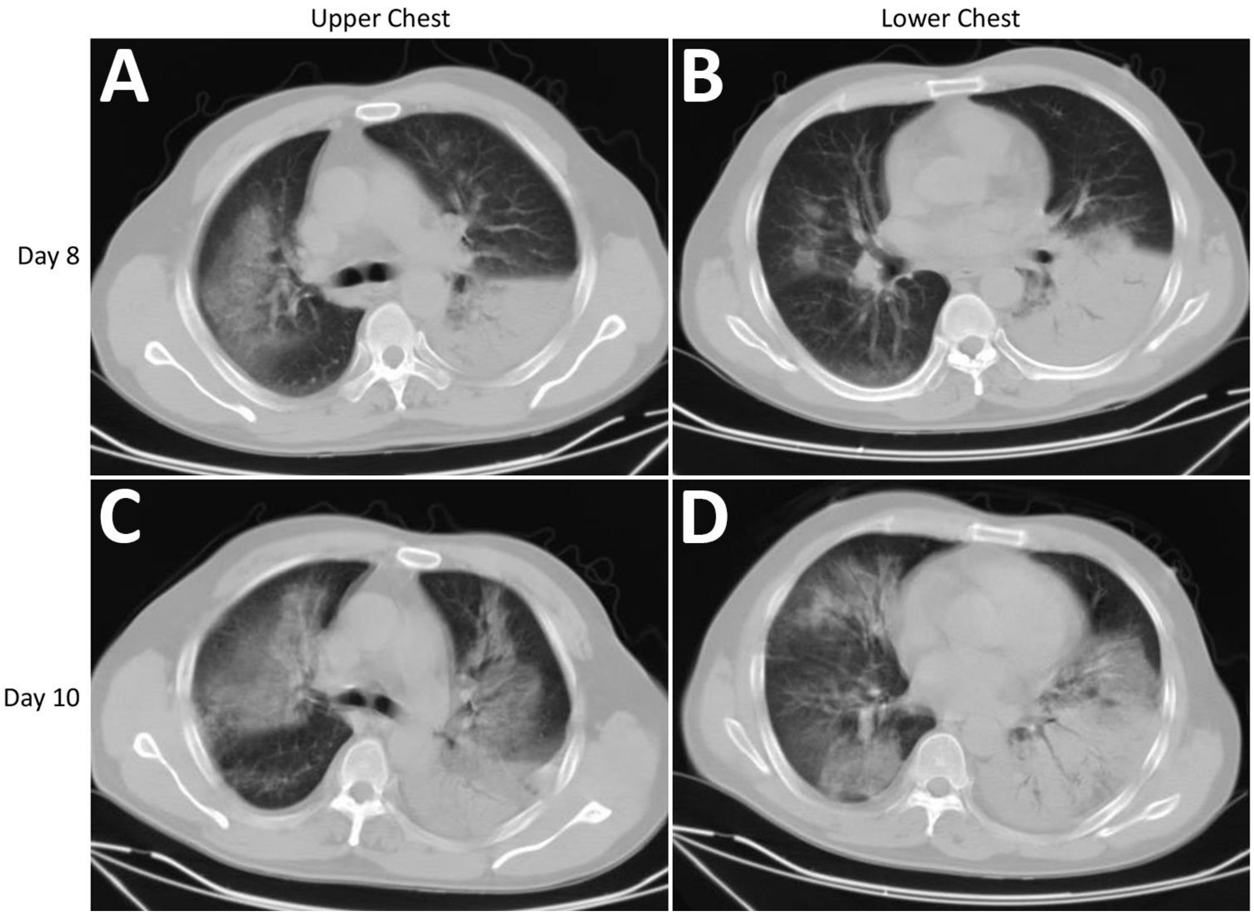


**Appendix Figure 1.** Chest computed tomography (CT) findings of case-patient AQ-1 who died from acute respiratory disease associated with human adenovirus 55 infection. A) and B) Upper and lower chest fields on April 22, day 8 after illness onset. Transverse CT images in the parenchymal window show ill-defined patchy and ground-glass opacities in all lobes of right lung and upper lobe of left lung and consolidation of lower lobe in left lung, suggestive of inflammatory exudation changes. Air-bronchogram can be found within the consolidation area. Note the slightly thickened right-sided pleura. C) and D) Upper and lower chest fields on the case-patient on April 24, day 10 after illness onset. The corresponding transverse CT images in the parenchymal window demonstrate increased areas of patchy opacities and consolidation in the bilateral lungs, as well as more obviously thickened right-sided pleura, indicative of the progression of disease.

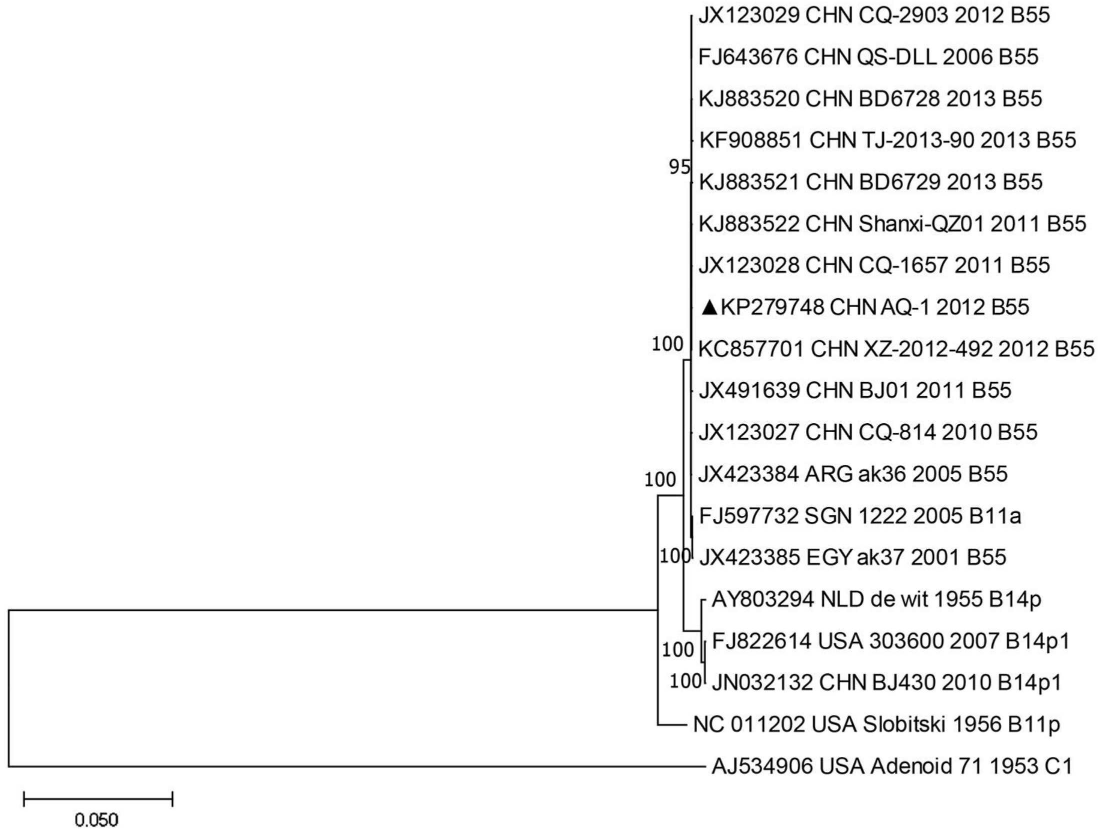


**Appendix Figure 2.** Phylogenetic analysis of human adenovirus 55 (HAdV-55) strain AQ-1_2012 from a fatal case of household transmission of HAdV-55, Anhui Province, China, 2012. Nucleotide sequences of archived HAdV-55 genomes retrieved from GenBank (https://www.ncbi.nlm.nih.gov/genbank) are used for comparison. Sequence names include GenBank accession number, isolation country, strain name, and year of isolation. Phylogenetic trees were constructed in MEGA7.1.0 (https://www.megasoftware.net) by using maximum-composite-likelihood method and applying default parameters with 1,000 bootstrap replications. Bootstrap proportions are shown as numbers on branches and at nodes. HAdV-C1 is included as an outgroup. Scale bar indicates number of nucleotide substitutions per site; ▲, indicates sequence from the case referenced.
